# Reproducibility of a Short Food Frequency Questionnaire for Japanese General Population

**DOI:** 10.2188/jea.17.100

**Published:** 2007-06-02

**Authors:** Nahomi Imaeda, Chiho Goto, Yuko Tokudome, Kaoru Hirose, Kazuo Tajima, Shinkan Tokudome

**Affiliations:** 1 Department of Food Science and Nutrition, Faculty of Human Life and Environmental Sciences, Nagoya Women’s University:; 2 Department of Health and Nutrition, School of Health and Human Life, Nagoya-bunri University:; 3 Department of Planning and Information, Aichi Prefectural Institute of Public Health.; 4 Division of Epidemiology and Prevention, Aichi Cancer Center Research Institute:; 5 Department of Health Promotion and Preventive Medicine, Nagoya City University Graduate School of Medical Sciences.

**Keywords:** food frequency questionnaire, diet assessment method, one-year interval reproducibility, middle-aged and elderly Japanese population

## Abstract

**BACKGROUND:**

In epidemiologic field studies, a food frequency questionnaire (FFQ) is one of the most feasible tools to assess usual dietary habits. The purpose of this study is to evaluate the reproducibility of consumption of foods and nutrients assessed with a self-administered short FFQ in a Japanese general population.

**METHODS:**

We have investigated 1-year interval reproducibility of a self-administered short FFQ, comprising 47 food items, and 8 frequency categories, among 1,918 subjects (844 males and 1,074 females) who participated in health check-up programs in Central Japan.

**RESULTS:**

Intakes of energy and 24 nutrients along with 15 food groups estimated using the first questionnaire (FFQ1) were approximately equal to those using the second (FFQ2). Spearman’s rank correlation coefficients (CCs) between intakes of nutrients quantified with FFQ1 and FFQ2 in males were distributed as 0.74 - 0.66- 0.55 (maximum - median - minimum), and intraclass CCs (ICCs) as 0.85 - 0.78 - 0.67. Among females, Spearman’s rank CCs were distributed as 0.73 - 0.62 - 0.54, and ICCs as 0.84 - 0.77 - 0.69. Percentages of exact agreement, exact agreement plus agreement within adjacent categories and disagreement according to quintile categorization were 43%, 80%, and 1%, for males, and 42%, 79%, and 1% for females. Reproducibility figures were higher for the elderly than for young people in both sexes.

**CONCLUSIONS:**

Our FFQ yielded substantially high reproducibility and it may be applicable for assessing consumption of foods/food groups and energy and selected nutrients for the middle-aged and elderly population in Japan.

There has been increasing interest in lifestyle, including dietary habits, as an etiological factor for chronic diseases. To establish strategies for lifestyle alterations, we need to adopt a comprehensive approach for evaluating dietary habits, alcohol consumption, smoking, physical exercise, and stress. However, Japanese dietary patterns differ from those of Western developed countries, due to its distinctive culture, climate, food supply system, cooking methods, and standard serving sizes.^1^^-^^3^ Japanese cuisine is rich in variety; for example, the major contributors of protein are rice, soybeans, and fish rather than meat and eggs. Moreover, people often enjoy not only Japanese foods but also Chinese, American, Italian, and French foods.

Food frequency questionnaires (FFQs) are generally accepted to be appropriate for ranking individuals according to consumption of foods and nutrients in large epidemiologic studies.^4^^,^^5^ When dealing with dietary data assessed with FFQs, validity and reproducibility are of note. Many articles concerning reproducibility of intake of foods and nutrients have been reported using various types of questionnaires, study subjects and time frames. Because most epidemiologic studies are based on FFQs with more than 100 items, subjects are forced to concentrate their attention for almost 1 hour. Therefore, we have evolved a self-administered short FFQ with only 47 items for dietary studies of the middle-aged and elderly general Japanese population.^6^

The present study aimed to explore reproducibility of a short FFQ with 47 items to elucidate whether it provides accurate information about full range of foods and nutrients. For this purpose, consumption of 15 foods and energy and 24 macro- and micro-nutrients was measured with the FFQs administered at a one-year interval to middle-aged and elderly Japanese.

## METHODS

Subjects were healthy members of the general population who participated in annual health check-ups at worksites or community centers in Aichi Prefecture, Central Japan, in 2002 and 2003. Of 3,828 subjects who had the health check-ups in the first year, 2,357 subjects were repeat participants of the next year. In our survey, registered nurses or public health nurses carried out interviews to fill-in missing information. After excluding 171 males and 86 females who had missing values in FFQs, or 3 males whose consumption was estimated less than 1000 kcal/day (4,184 J/day), or 17 females whose consumption was those 800 kcal/day (3,347 J/day), and 162 people who reported changing their diet before the second FFQ due to their health condition, finally we included 1,918 (844 males and 1,074 females, 23-86 years old) who gave informed consent to the present study.

Intake of 15 foods/food groups and energy, 24 macro- and micro-nutrients was assessed in 2002 (hereafter FFQ1) and in 2003 (hereafter FFQ2). The nutrients were protein, fat, carbohydrate, minerals (potassium, calcium, and iron), vitamins (carotene, vitamins A, D, E, B_1_, B_2_, folate, and C) and total dietary fiber (TDF) (soluble DF and insoluble DF). Fat was divided into saturated fatty acids, monounsaturated fatty acids, n-6 and n-3 polyunsaturated fatty acids (PUFAs), and n-3 highly-unsaturated fatty acids (n-3 HUFAs, including icosapentaenoic acid (IPA, 20:5), docosapentaenoic acid (DPA, 22:5) and docosahexaenoic acid (DHA, 22:6)) and cholesterol. Consumption of foods (grams/day) and nutrients was calculated using typical/standard values from the literature.^7^^,^^8^

First, we compared average daily intake for foods/food groups, energy, and macro- and micro-nutrients according to the FFQ1 and FFQ2. Differences were expressed as percentage values after each value was logarithmically transformed and adjusted for total energy, to allow calculation of intra-class correlation coefficients (hereafter ICCs), and Spearman’s rank correlation coefficients (hereafter CCs) for intake of selected foods and nutrients between the two FFQs.^9^^-^^13^ Furthermore, we compared the ICCs by 10-year age group.

Dividing intakes of foods and nutrients into quintiles based on the FFQ1 and FFQ2, we calculated the degree of misclassification across the quintiles as follows: the proportions categorized into the same quintiles, those categorized into the same quintiles plus adjacent quintiles, or those categorized into the opposite quintiles.

All statistical analyses were performed using SPSS^®^ version 12.0.

Instructions about the purpose of the present study were noted at the top of the questionnaire. We obtained informed consent from participants. The protocol was approved by the Ethical Review Committee of the Nagoya City University Graduate School of Medical Sciences.

## RESULTS

### Characteristics of study subjects

Mean ± standard deviation (SD) values for age were 56.6 ± 13.4 years for males and 57.0 ± 10.1 for females. At baseline, the body mass index (BMI: kg/m^2^) were 23.3 ± 2.7 for males and 22.2 ± 2.9 for females, and no changes were evident at the second survey. According to the distribution for BMI by age, the percentages of overweight individuals (BMI=25+) were distributed from 22% to 34% by age group in males. The percentage of underweight (BMI<18.5) individuals under 40 years of age was 17% and those overweight accounted for only 4% in females.

### Intake of foods/food groups

Table 1 shows comparisons between the average daily intake of foods/food groups according to the FFQ1 and FFQ2. The values were approximately equal at both time points. Spearman’s rank CCs with energy-adjustment (maximum-median-minimum) were distributed as 0.80 (alcohol) - 0.65 - 0.57 (green tea, coffee) for males, and 0.69 (rice) - 0.60 - 0.54 (oils) for females. ICCs with log-transformation and energy-adjustment were distributed as 0.87 (alcohol) - 0.78 - 0.68 (green tea) for males, and 0.85 (alcohol) - 0.76 - 0.69 (rice) for females.

**Table 1.** Comparison of mean daily intakes of foods/food groups and correlation coefficients (CCs) with food frequency questionnaire 1 (FFQ1) and FFQ2.

Food group	Males (n= 844)					Females (n=1,074)				
	
	FFQ1	FFQ2		FFQ1 vs. FFQ2		FFQ1	FFQ2		FFQ1 vs. FFQ2	
					
	Consumption (g/day)	Consumption (g/day)	% difference*	Spearman’s rank CC	ICC**	Consumption (g/day)	Consumption (g/day)	% difference*	Spearman’s rank CC	ICC**
Rice (cooked)	524 ± 216	508 ± 215	-3	0.65	0.80	334 ± 108	327 ± 112	-2	0.69	0.69
Bread, noodles and potatoes	112 ± 77	118 ± 74	5	0.60	0.71	104 ± 59	106 ± 59	2	0.60	0.70
Soybean and soybean products	85 ± 51	83 ± 50	-2	0.71	0.81	90 ± 47	90 ± 49	0	0.65	0.79
Green-yellow vegetables	63 ± 51	64 ± 51	2	0.63	0.78	83 ± 54	85 ± 60	2	0.62	0.77
Other vegetables and seaweed	71 ± 50	72 ± 46	2	0.65	0.78	100 ± 56	100 ± 59	0	0.56	0.72
Fruit	56 ± 53	54 ± 54	-3	0.66	0.82	81 ± 63	80 ± 68	-1	0.66	0.76
Fish and other seafoods	57 ± 36	60 ± 37	4	0.66	0.80	58 ± 31	59 ± 33	2	0.65	0.79
Meat	34 ± 22	35 ± 23	4	0.59	0.78	34 ± 19	33 ± 22	-2	0.57	0.83
Eggs	22 ± 17	22 ± 16	1	0.66	0.76	23 ± 14	22 ± 14	-4	0.57	0.73
Dairy products	112 ± 105	113 ± 100	0	0.75	0.83	159 ± 111	164 ± 115	4	0.69	0.83
Oil	16 ± 10	16 ± 10	3	0.59	0.73	18 ± 9	17 ± 11	-4	0.54	0.71
Confectioneries	17 ± 22	17 ± 17	-1	0.60	0.73	25 ± 22	24 ± 22	-5	0.62	0.76
Green tea	343 ± 254	339 ± 254	-1	0.57	0.68	423 ± 218	431 ± 215	2	0.59	0.75
Coffee	156 ± 115	154 ± 116	-1	0.57	0.69	211 ± 109	216 ±108	2	0.59	0.76
Alcohol beverage	118 ± 140	128 ± 158	8	0.80	0.87	16 ± 50	18 ± 56	11	0.56	0.85

Median				0.65	0.78				0.60	0.76

* : (FFQ2-FFQ1)/FFQ1 (%)

**: intraclass correlation coefficient

Consumption (grams per day) are shown as mean ± standard deviation.

ICCs for nutrients were calculated after values were log-transformed and energy-adjusted.

For males, percentages of exact agreement with energy-adjustment were distributed as 59% (alcohol) - 44% - 41% (oil, other vegetables and seaweed), and exact agreement plus agreement within adjacent categories were 91% (alcohol) - 80% - 75% (meat), the median for disagreement was 1% (Table 2). For females, percentages of exact agreement with energy-adjustment were distributed as 46% (rice, green tea) - 41% - 38% (oil), exact agreement plus agreement within adjacent categories were distributed as 83% (rice, dairy products) - 78% - 74% (meat), and the median for disagreement was also 1%. We could not categorize alcohol intake into quintiles for females because 70% had no drinking habits.

**Table 2.** Level of agreement according to quintile classification of daily intake of foods/food groups based on food frequency questionnair 1 (FFQ1) and FFQ2 (%).

Food group	Males			Females		
	
	Agreement		Disagreement	Agreement		Disagreement
			
	Same quintiles	Same and +/- 1 quintiles	Opposite quintiles	Same quintiles	Same and +/- 1 quintiles	Opposite quintiles
Rice (cooked)	44	82	2	46	83	1
Bread, noodles and potatoes	43	77	2	41	80	2
Soybean and soybean products	44	83	1	41	80	1
Green-yellow vegetables	44	79	1	40	80	1
Other vegetables and seaweed	41	80	2	39	76	2
Fruit	44	80	1	39	80	1
Fish and other seafoods	45	80	1	42	79	1
Meat	41	75	1	39	74	1
Eggs	46	80	1	42	76	2
Dairy products	51	85	1	45	83	1
Oil	41	77	1	38	76	2
Confectioneries	43	78	2	42	78	1
Green tea	45	78	2	46	77	2
Coffee	45	78	2	45	77	2
Alcohol beverage	59	91	1	-	-	-

Median	44	80	1	41	78	1

Proportions for nutrients were calculated after intakes were energy-adjusted.

### Intake of energy, macro- and micro-nutrients

Table 3 lists crude values for daily intake of energy, macro- and micro-nutrients based on the FFQ1 and FFQ2. The differences were distributed from -4% to 4% and the intakes of foods and nutrients assessed with both FFQs were very similar to these of previous semi-quantitative FFQs with more than 100 items.^14^^,^^15^

**Table 3.** Comparison of mean daily intakes of selected nutrients and correlation coefficients (CCs) with food frequency questionnaire 1 (FFQ1) and FFQ2.

Nutrient	Males					Females				
	
	FFQ1	FFQ2		FFQ1 vs. FFQ2		FFQ1	FFQ2		FFQ1 vs. FFQ2	
					
	Consumption (g/day)	Consumption (g/day)	% difference*	Spearman’s rank CC	ICC**	Consumption (g/day)	Consumption (g/day)	% difference*	Spearman’s rank CC	ICC**
Energy (MJ)	8.3 ± 1.8	8.2 ± 1.8	-1	0.71	0.84	6.8 ± 1.0	6.8 ± 1.0	-1	0.54	0.73
Protein (g)	61 ± 13	61 ± 13	1	0.63	0.77	55 ± 10	55 ± 11	0	0.60	0.75
Fat (g)	44 ± 12	44 ± 12	2	0.67	0.80	46 ± 11	45 ± 12	-2	0.64	0.78
Carbohydrate (g)	311 ± 85	306 ± 84	-1	0.67	0.79	235 ± 41	232 ± 45	-1	0.67	0.78

Potassium (mg)	2,290 ± 591	2,270 ± 580	-1	0.70	0.84	2,442 ± 559	2,449 ± 602	0	0.65	0.80
Calcium (mg)	532 ± 163	537 ± 157	1	0.71	0.84	602 ± 162	616 ± 174	2	0.66	0.81
Iron (mg)	7.5 ± 2.4	7.5 ± 2.4	0	0.71	0.84	8.2 ± 2.1	8.2 ± 2.2	0	0.68	0.81

Carotenes (mg)	2,951 ± 1,465	3,006 ± 1,481	2	0.63	0.78	3,525 ± 1,539	3,594 ± 1,743	2	0.62	0.77
Vitamin A (µg)	966 ± 506	1,008 ± 575	4	0.57	0.70	979 ± 421	1,021 ± 516	4	0.60	0.73
Vitamin D (µg)	8 ± 4	8 ± 4	4	0.66	0.80	8 ± 3	8 ± 4	2	0.63	0.77
Vitamin E (mg)	8.1 ± 2.4	8.3 ± 2.3	2	0.60	0.74	8.8 ± 2.2	8.8 ± 2.4	0	0.60	0.76
Vitamin B_1_ (mg)	0.7 ± 0.1	0.7 ± 0.1	1	0.55	0.67	0.6 ± 0.1	0.6 ± 0.1	0	0.55	0.69
Vitamin B_2_ (mg)	1.1 ± 0.3	1.1 ± 0.3	1	0.67	0.80	1.2 ± 0.3	1.2 ± 0.3	1	0.63	0.78
Folate (µg)	331 ± 114	334 ± 116	1	0.63	0.78	378 ± 117	383 ± 124	1	0.62	0.76
Vitamin C (mg)	93 ± 36	93 ± 36	0	0.67	0.80	118 ± 42	117 ± 42	-1	0.66	0.80

Total dietary fiber (g)	10.9 ± 3.6	11 ± 3.6	1	0.72	0.85	12.4 ± 3.7	12.5 ± 4.0	0	0.70	0.83
Insoluble dietary fiber (g)	7.7 ± 2.5	7.8 ± 2.5	1	0.73	0.85	9.0 ± 2.6	9.0 ± 2.8	0	0.73	0.84
Soluble dietary fiber (g)	2.0 ± 0.7	2.0 ± 0.7	1	0.74	0.85	2.2 ± 0.7	2.3 ± 0.8	1	0.71	0.83

Cholesterol (mg)	249 ± 76	250 ± 74	0	0.66	0.78	254 ± 66	250 ± 67	-2	0.60	0.75
Saturated fatty acids (g)	11.1 ± 2.6	11.1 ± 2.5	0	0.65	0.78	11.7 ± 2.7	11.6 ± 2.7	-1	0.66	0.80
Monounsaturated fatty acids (g)	16.2 ± 4.5	16.5 ± 4.4	2	0.60	0.73	16.7 ± 3.9	16.5 ± 4.4	-2	0.56	0.72
Polyunsaturated fatty acids (g)	13.6 ± 3.9	13.7 ± 3.8	1	0.57	0.72	14.2 ± 3.5	14.1 ± 3.9	-1	0.55	0.73
n-6 Polyunsaturated fatty acids (g)	2.3 ± 0.7	2.4 ± 0.7	2	0.59	0.74	2.4 ± 0.6	2.4 ± 0.6	-1	0.59	0.74
n-3 Polyunsaturated fatty acids (g)	11.3 ± 3.3	11.4 ± 3.3	1	0.56	0.70	11.8 ± 3.1	11.6 ± 3.3	-1	0.56	0.73
n-3 Highly-unsaturated fatty acids (g)	0.8 ± 0.4	0.8 ± 0.4	-4	0.60	0.77	0.8 ± 0.4	0.8 ± 0.3	-2	0.56	0.73

Median				0.66	0.79				0.62	0.77

* : (FFQ2-FFQ1)/FFQ1 (%)

** : intraclass correlation coefficient

Daily consumption is shown as mean±standard deviation.

ICCs for nutrients were calculated after values were log-transfomied and energy-adjusted.

When the values of nutrients intakes were energy-adjusted, Spearman’s rank CCs were distributed as 0.74 (soluble DF) - 0.66 - 0.55 (vitamin B1) for males, and 0.73 (insoluble DF) - 0.62 - 0.54 (energy) for females. ICCs were distributed as 0.85 (total DF, insoluble DF, soluble DF) - 0.78 - 0.67 (vitamin B_1_) for males, and 0.84 (insoluble DF) - 0.77 - 0.69 (vitamin B_1_) for females. For both sexes, the Spearman’s rank CCs and ICCs for calcium, iron, and dietary fiber were high.

Percentages of exact agreement were distributed as 47% (vitamin D, soluble DF) - 43% - 37% (vitamin B_1_, n-3PUFAs), exact agreement plus agreement with adjacent categories as 85% (insoluble DF) - 80% - 75% (vitamin B_1_, PUFAs, n-3PUFAs), and disagreement were distributed as 1-2% in males (Table 4). For females, the respective percentages were 44% (calcium, total DF, insoluble DF, soluble DF) - 42% - 34% (PUFAs), 84% (total DF, soluble DF) - 79% - 74% (vitamin B_1_, PUFAs, n-3PUFAs, n-3HUFAs), and 0% (total DF, insoluble DF) - 1% - 3% (energy).

**Table 4.** Level of agreement and disagreement according to quintile classification of daily intake of selected nutrients based on food frequency questionnaire 1 (FFQ1) and FFQ2 (%).

Nutrient	Males			Females		
	
	Agreement		Disagreement	Agreement		Disagreement
			
	Same quintiles	Same and +/- 1 quintiles	Opposite quintiles	Same quintiles	Same and +/- 1 quintiles	Opposite quintiles
Energy	45	83	1	39	76	3
Protein	41	80	1	38	79	1
Fat	43	80	1	41	78	1
Carbohydrate	44	82	1	43	81	1

Potassium	44	82	1	42	80	1
Calcium	43	83	1	44	81	1
Iron	40	83	1	42	81	1
Carotenes	42	79	2	39	79	1

Vitamin A	44	77	2	41	79	2
Vitamin D	47	81	1	42	78	1
Vitamin E	40	78	2	36	75	1
Vitamin B_1_	37	75	2	37	74	2
Vitamin B_2_	45	79	1	42	80	1
Folate	42	78	1	40	79	1
Vitamin C	43	81	1	43	79	1

Total dietary fiber	44	84	1	44	84	0
Insoluble dietary fiber	46	85	1	44	83	0
Soluble dietary fiber	47	84	1	44	84	1

Cholesterol	45	80	1	42	79	2
Saturated fatty acids	43	80	1	43	80	1
Monounsaturated fatty acids	42	77	1	35	76	1
Polyunsaturated fatty acids	40	75	1	34	74	2
n-6 polyunsaturated fatty acids	40	77	1	36	77	1
n-3 polyunsaturated fatty acids	37	75	1	36	74	2
n-3 highly-unsaturated fatty acids	44	78	2	43	74	1

Median	43	80	1	42	79	1

Proportions for nutrients were calculated after intakes were energy-adjusted.

The proportions categorized into the same quintiles, those categorized into the same quintiles plus adjacent quintiles, or those categorized into the opposite quintiles.

### Reproducibility by age

As a whole, no significant differences were observed in reproducibility indices for foods/food groups across age groups (data not shown). The median values of ICCs were more than 0.73 for foods/food groups by age group in both sexes. Figures for people aged 50 years or older were generally high for both sexes, and values for the group over 70 years were highest at 0.79 for males and 0.78 for females. With respect to macro- and micro-nutrients, the median indices were more than 0.70 for both sexes. Figures for the group aged over 70 years, in particular, exhibited the highest value of 0.82 for males and 0.78 for females. Those values for most nutrients, however, in males under 40 years of age, were rather lower than those for over 70 years (p<0.01). Figures for energy intake among females under 40 years of age were somewhat lower than for other age groups. Accordingly, this FFQ yielded equivalent or higher reproducibility values compared with the full version of semi-quantitative FFQ administered to Japanese female dietitians.^15^

## DISCUSSION

We formerly observed fairly high validity values for consumption of energy and macro- and micro-nutrients assessed with our questionnaire versus 3-day weighed diet records.^16^ We also detected moderate validity between intake of fatty acids estimated with this questionnaire against plasma concentration.^17^ In the present study, we observed substantially high one-year interval reproducibility value for the respective foods and nutrients assessed with the FFQ administered to middle-aged and elderly Japanese people. Median indices of Spearman’s rank CCs for foods/food groups were greater than 0.60, the median ICC figures being greater than 0.76 for both sexes. The median Spearman’s rank CCs for macro- and micro-nutrients were greater than 0.62, and the median ICC values were greater than 0.77 for both sexes.

Furthermore, we paid special attention to the differences in reproducibility indices by age group and observed slightly lower reproducibility values in young males under 40 years of age than in other age groups. This probably reflects their wide selection of foods/food groups and their active and free lifestyle. On the other hand, contrary to the report of Shimizu et al,^18^ higher reproducibility values were noted in elderly people, which might be expected due to the fact that they lead rather traditional and ordinary lives, including dietary habits.^19^

Although women are generally more interested in the foods they eat and cook than men, there were no remarkable differences in reproducibility figures for foods and nutrients between sexes in the present study. Instead, as a whole, the reproducibility values for males were rather higher than those for females. The indices for the young generation under 39 years of age, in particular, were somewhat lower for consumption of staple foods, including rice, noodles and bread along with energy, presumably because women in the young generation are keen on diet to keep in shape.

We compared our one-year interval reproducibility values for foods/food groups and macro- and micro-nutrients with those indices of Japanese short FFQs, including approximately 50 items of foods/food groups (Table 5).^11^^-^^13^ The median Spearman’s rank CCs for foods and nutrients distributed between 0.49 and 0.57 in both sexes. Our reproducibility figures were 10% on average higher than those with smaller minimum-maximum ranges, which may be partly due to the fact that the number of subjects in this survey was greater than those in the recent literature.^11^^-^^13^

**Table 5.** Comparison of one-year interval reproducibility indices of Japanese short food frequency questionnaires.

Authors	Year	No. of food items	Sex	n	Median (range) of Spearman’s rank CCs	

					Food groups	Nutrients
Ogawa K et al^11^	2003	40	Male	55	0.50 (0.30-0.70)	0.49 (0.31-0.71)
			Female	58	0.57 (0.39-0.66)	0.50 (0.40-0.64)

Sasaki S et al^12^	2003	44	Male	101	0.50 (0.38-0.71)	0.49 (0.30-0.82)
			Female	108	0.49 (0.30-0.74)	0.50 (0.32-0.68)

Ishihara J et al^13^	2003	44	Male	143	0.51 (0.33-0.72)	0.57 (0.39-0.77)
			Female	146	0.50 (0.40-0.80)	0.54 (0.38-0.70)

Present study (2007)	2007	47	Male	844	0.65 (0.57-0.80)	0.66 (0.55-0.74)
			Female	1,074	0.60 (0.69-0.54)	0.62 (0.54-0.73)

CC: correlation coefficient

In conclusion, we previously observed fairly high relative validity values for consumption of foods and nutrients estimated with our short FFQ versus those assessed with 3-day weighed diet records.^14^ Moderate validity was attained for intake of fatty acids measured with our FFQ against plasma concentration.^15^ The present study detected substantially high one-year interval reproducibility values for consumption of foods and nutrients assessed with our FFQ. The abbreviated questionnaire requires less time to fill out and would thus be applicable to a middle-age and elderly general populace for assessing usual dietary habits.
